# Portrayals of Pain in Children's Popular Media: Mothers' and Fathers' Beliefs and Attitudes

**DOI:** 10.3389/fpain.2022.898855

**Published:** 2022-05-06

**Authors:** Maria Pavlova, Kendra Mueri, Madison Kennedy, Sarah Wallwork, G. Lorimer Moseley, Abbie Jordan, Melanie Noel

**Affiliations:** ^1^Department of Psychology, University of Calgary, Calgary, AB, Canada; ^2^School of Public Health, University of Alberta, Edmonton, AB, Canada; ^3^IIMPACT in Health, University of South Australia, Adelaide, SA, Australia; ^4^Department of Psychology and Centre for Pain Research, University of Bath, Bath, United Kingdom; ^5^Alberta Children's Hospital Research Institute, Calgary, AB, Canada; ^6^Hotchkiss Brain Institute, Calgary, AB, Canada; ^7^Mathison Centre for Mental Health Research and Education, Calgary, AB, Canada

**Keywords:** pain portrayal, thematic analysis, mothers, fathers, pediatric pain

## Abstract

Evidence suggests that children's popular media may model maladaptive and distorted experiences of pain to young children. In a recent study, pain depicted in popular media targeting 4–6-year-olds was frequently and unrealistically portrayed, evoked little response or empathy from observing characters, and perpetuated unhelpful gender stereotypes. Parents play a critical role in both children's pain experiences and children's media consumption. Yet, no study to date has examined parents' beliefs and attitudes regarding how pain is portrayed in media for young children. The present study aimed to fill this gap by examining how parents perceive and appraise painful instances depicted in children's popular media. Sixty parents (48% fathers) of children aged 4 to 6 years completed a semi-structured interview to assess their general beliefs and attitudes toward how pain is portrayed in children's media. Inductive reflexive thematic analysis was conducted to identify and analyze key patterns in the data. Qualitative analyses generated two major themes representing parental beliefs regarding pain that is portrayed in children's media: “entertaining pain” and “valuable lessons”. Findings reveal that parents believe that pain portrayed in popular media serves either a function of entertaining and amusing children or can provide valuable lessons about appropriate emotional responses and empathic reactions. Further, pain portrayals could also instill valuable lessons and provide children with a point of reference and language for their own painful experiences. Parents serve as a primary socialization agent for young children; thus, it is important that parents remain aware of underlying messages about how pain is portrayed in children's popular media so that they can optimally discuss these portrayals, promote their children's pain education and understanding and positively impact future pain experiences.

## Introduction

Our response to noxious stimuli begins developing from birth. Painful medical procedures, immunization injections, accidental injures (e.g., breaking a limb, concussion), and everyday injuries that result in bumps and bruises provide children with first-hand experiences with sensory and affective dimensions of pain. Pain is also inherently a social experience (1). Children's responses to pain depend on who is present during the painful experience and how those observers respond to sufferers' pain (2). Parents play a critical role in children's pain experiences. Parental verbal and nonverbal responses and reactions during needle procedures (e.g., venipunctures, vaccine injections) can exacerbate children's pain (3). The ways in which young children and parents talk about previous painful experiences impacts how children remember their pain (4) and is associated with children's empathic concern toward another person's pain (5). Beyond parent-child interactions, undoubtedly children's understanding and responses to pain are also shaped by broader socio-cultural influences, such as popular media. However, no studies have examined parental beliefs and attitudes toward how pain is portrayed in children's television shows and movies.

Indeed, popular media (e.g., television shows, movies, video games, books) have been argued to be a powerful source for children's learning (6–8). Children's everyday lives are permeated with various media formats that children either directly attend to or are present in the background. By the age of 4 years, children usually have their own mobile device, in addition to access to television, and look at screens daily for nearly 2 hours (9). Ninety percent of young children (i.e., aged 2 to 5 years) exceed the American Academy of Pediatrics recommendation of watching no more than 1 h of television per day (10). Consequently, popular media saturates children's environments and becomes a critical source of children's socialization, influencing their attitudes, beliefs, and behaviors (7). Children attend to, remember, and learn a multitude of things from children's media. For example, having watched video clips that contained stereotypical portrayals of gender stereotypes (i.e., boys demonstrating higher math performance than girls) school-aged boys and girls reported higher endorsement of gender stereotypes (11). It is likely that children similarly attend to, and learn from, media portrayals of pain.

Pain is commonly portrayed in children's media (12) although parents think that it is rarely depicted in sources like movies, books, television shows, textbooks (13) Yet, a recent study demonstrated that instances of pain are frequently portrayed in young children's popular media (i.e., television shows, movies) (12). Pain associated with violence and injury was the most common, whereas other types of pain (e.g., chronic, procedural) that are frequently experienced by children in real life, were infrequently portrayed. Pain instances amongst girl characters were grossly underrepresented, with boy characters accounting for 78% of all pain instances. Boy, vs. girl, characters expressed greater distress, but characters who were observing the sufferers more often expressed concern toward girl characters experiencing pain. Observing characters demonstrated little empathy for sufferers experiencing pain. A substantial proportion of observing characters (41%) witnessed but did not respond to the sufferers' pain (e.g., did not exhibit concern, did not help). The latter is particularly notable as empathy for pain follows a developmentally distinct trajectory from that of empathy for other negative emotions (e.g., sadness) (14). Young children reacted similarly to another person being sad; however, older children were observed to be more concerned about another person being physically hurt than younger children were.

Parents have expressed positive attitudes toward children's media more broadly, and endorsed media exposure starting at a young age (i.e., 0–3 years) as being beneficial for brain development and important for proficiency in using technology in the future (15). Further, the majority of parents disagreed that children should watch exclusively educational programs (15). Given the broadly unhelpful portrayals of pain in children's popular media (12), yet the broad endorsement of media exposure by parents, as well as the important role that parents play in shaping children's pain experiences, there is a pressing need to understand how parents perceive, appraise, and interpret painful experiences portrayed in children's media and how they discuss this with their child.

This study aimed to fill this knowledge gap by examining how parents perceive and discuss pain as depicted in popular children's media. Through an idiographic qualitative lens, the present study investigated how parents of young children make sense of, interpret, and appraise pain portrayals in the media (i.e., popular television shows and movies).

## Materials and Methods

### Participants

The convenience sample included 60 fathers and mothers who were recruited from the community in a large city in Western Canada. Community recruitment methods included bulletin board postings, postings on social media (i.e., Facebook, Instagram, Twitter), a research database of healthy children whose parents indicated interest in participating in research, and word of mouth. Participants were eligible for the study if they had a child aged 4–6 years and could speak/understand English. Participants were excluded if their child had developmental/language delays or mental health disorders (e.g., ADHD, ODD). On average, parents were ~38.8 years old (*SD* = 5.7) and 48% were fathers. The mean age of children was 5.4 years (*SD* = 0.8) and 48.3% were girls. Most parents reported their ethnic background as white (85%). Sociodemographic characteristics are summarized in Table 1.

**Table 1:** Sociodemographic characteristics of the sample.

**Sociodemographic characteristics**	***N* = 60**
Parent age (years), *M* (*SD*)	38.8 (5.7)
Parent gender (female, % and *n*)	51.7 (31)
Parent race (% and *n*)	
Aboriginal	1.7 (1)
Latin American	3.3 (2)
South Asian	1.7 (1)
South East Asian	1.7 (1)
White	85 (51)
Multiracial	1.7 (1)
Other	5 (3)
Child age (months), *M* (*SD*)	65.3 (9.4)
Child sex (female, % and *n*)	48.3 (29)
Child race (% and *n*)	
Aboriginal	1.7 (1)
South East Asian	1.7 (1)
White	74.6 (44)
Multiracial	13.6 (8)
Other	8.5 (5)
Parent highest level of education (% and *n*)	
High school or less	6.7 (4)
Vocational school/some college	6.7 (4)
College/Bachelor's degree	50 (30)
Graduate school	36.7 (22)
Work status (% and *n*)	
Full time	70 (42)
Part time	18.3 (11)
Not employed	11.7 (7)
Household annual income (% and *n*)	
<$10,000	1.7 (1)
$20,000–$29,000	1.7 (1)
$30,000–$39,000	0 (0)
$40,000–$49,000	0 (0)
$60,000–$69,000	3.3 (2)
More than $70,000	93.3 (56)
Parent marital status (% and *n*)	
Married	81.7 (49)
Common-law	8.3 (5)
Separated/divorced	10 (6)

### Procedure

All parents who expressed interest in participating in the study were screened via phone or email to determine eligibility. Once eligibility was determined, participants were invited to come to a laboratory based at the University to participate in the study. At the beginning of the visit, parents provided informed consent. Next, parents and children, with a researcher present in the room, watched a series of ten counterbalanced clips illustrating painful events in children's popular television shows and movies. Next, parents and children, without the researcher in the room, discussed the clips. Parent and children were asked to discuss the clips as normally as would talk about movies/television shows that they watched together in their everyday lives; no time limit was given. These clips were selected by the researchers from a sample of 454 pain instances that were previously extracted from a cross-section of children's popular media (12). This cross-section included 10 popular movies and six television shows targeted toward young children (i.e., 4- to 6-year-olds) (12). The researchers aimed to select a wide scope of clips depicting pain that were associated with various types of events (e.g., injury, fall, scratch) (16, 17), an approximately even distribution of character genders (i.e., male, female, and non-binary), and an approximately equal number of television shows and movies. Once parents and children finished watching the clips, parents completed a brief semi-structured interview designed to assess parental beliefs and attitudes toward pain depicted in children's media. Parent interviews were audio-recorded and transcribed verbatim. Once the interview was completed, parents filled out a brief sociodemographic questionnaire. At the end of the study, participants received a $20 CAD gift card in recognition of their time and participation.

### Measures

#### Sociodemographics

Parents reported their age, race/ethnicity, relationship to the child, household income, education level, as well as their children's age, sex, and race/ethnicity.

#### Parent Interview

A brief six-question semi-structured interview was used to elicit parents' general perceptions, beliefs, and attitudes toward how pain is portrayed in children's popular media (see Table 2). The interview schedule comprised six open-ended questions with the aim of eliciting honest and natural responses from parents. The interview questions were directed to obtain parents' thoughts on how pain is portrayed in media that their children commonly consumed, as well as the video clips that parents and children watched prior to the interview. The interview questions covered different types of pain that media characters encountered, and how parents believe media influences the broader public's perceptions of pain, particularly pain experienced by young children (e.g., *In your experience, how is physical pain portrayed in children's movies and TV shows?*). Open-ended prompts (e.g., *If so, how?*) were used to facilitate in-depth answers. The questions were based on a previous examination of pain in children's popular media (12). The interviews, on average, lasted 9.5 min (*SD* = 4.5, *range* 3.5–28.0 min).

**Table 2:** Parental beliefs about pain portrayal: semi-structured interview questions.

1.	In your experience, how has physical pain been portrayed in children's movies and TV shows? a) Have you seen injuries portrayed in kids' shows? If yes, how has it been portrayed? b) Have you seen procedural pain, like needles or vaccinations, depicted in kids' shows? If yes, how has that been portrayed? c) Have you seen things like stomach aches or headaches shown in children's shows? If so, how has that been shown?
2.	How is pain portrayed in girl characters vs. boy characters? Do you find there is a difference? If so, what have you noticed?
3.	What do you think this is teaching kids about pain/what are they learning?
4.	Does this influence how they experience pain? If so, how?
5.	How do you think pain *should* be portrayed in children's media?

### Analyses

Inductive reflexive thematic analysis is a theoretically flexible approach which facilitates the identification and analysis of thematic patterns across participant accounts (18). Induction reflexive thematic analysis has been previously used in pediatric pain research to examine interview data (19–21). Transcripts from the interviews were uploaded and qualitatively analyzed with QSR International's NVivo 12 software by a primary coder (MP). The analysis primarily focused on semantic features within the data (e.g., focusing on the content and meaning of what participants shared) rather than latent features (e.g., how participants spoke). Interview transcripts were continually and actively read to familiarize with the data. An iterative qualitative approach was used, such that potential themes were continuously revised to determine the final major themes. First, candidate themes were generated. Upon reflection and further data analysis, five candidate themes were collapsed into two themes to avoid content and theoretical overlap. Throughout the inductive reflexive thematic analysis, consultation and feedback were sought from within the study team (MN, MP, KM, AJ who is a researcher with over 16 years of experience in conducting qualitative analyses) to discuss thematic development. The following Braun & Clarke's criteria (22) for the quality of thematic analysis were met: (1) interview transcripts were checked against the original audio recordings to confirm accuracy; (2) each data item was given equal attention; (3) quotations were balanced with analytic narrative; (4) the researchers were positioned as active in the analytic process (e.g., MP kept a reflexive journal). All authors reviewed and agreed upon the final themes.

The quantitative data were analyzed using the SPSS version 26.0. Descriptive analyses and frequency statistics were conducted to report sociodemographic characteristics of the sample.

## Results

Inductive reflexive thematic analysis of the data generated two themes that represent parental opinions regarding pain as portrayed in children's popular media. Each theme is supported with anonymized quotations from participants that provide evidence for the accompanying interpretation. Participants' names were replaced with pseudonyms. Themes comprise: “entertaining pain” and “valuable lessons” and each will be presented in turn below.

### Theme One: Entertaining Pain

The first theme captures parents' impressions that pain, as portrayed in children's media, was used to amuse children, as well as to capture their attention. To achieve these goals, pain was often portrayed as comedic, unrealistic, and exaggerated to the point of having no connection with pain experienced in real daily life. For example, one parent described how in children's shows “*pain doesn't hurt”* (Kate, mother). Parents noted two key characteristics of entertaining pain: (1) pain was portrayed as fleeting and having no consequences; (2) pain was portrayed as comedic.

First, parents expressed that pain in children's media could be entertaining because it was shown as momentary and having no short- or long-lasting consequences. According to some parents, pain was transitory as it simply stopped and/or could be easily and quickly fixed. One parent commented that media's portrayal of pain “*removes the emotion out of it”* (James, father). Thus, pain that was portrayed in the media lacks a crucial aspect of real-life pain experiences—the affective aspect that includes suffering and emotional reactions (e.g., surprise, fear, anger) to pain. Pain as portrayed in media was perceived as occurring and immediately dissipating, leaving no physical or emotional trace.

The unrealistic portrayal of pain, being immediate and resulting in a swift and uncomplicated recovery, along with the absence of pain-related emotional suffering, served as a marker of the character's wellbeing. Further, the absence of consequences and emotions, coupled with an easy recovery, allowed pain to be treated as a plot mechanism propelling the story, as opposed to a part of the story that required attention and (empathetic) reactions from other characters (and the audience). In media, the function of pain was to facilitate the flow of the story; pain was unimportant and not worthy of attention in its own right.

“*[Pain is] typically [used] as a plot tool. Either to move the plot from A to B, or sometimes to inject humour into a situation”*. (Paul, father)

The second part of Paul's comment addresses another reason why pain, according to parents, was entertaining: pain was portrayed as silly and comedic, a “*slapstick comedy”* (Norah, mother). The comedic portrayal of pain identified the social aspect of pain as a complex, multidimensional experience that involves the victim/sufferer *and* people witnessing that person in pain. In everyday life, social response to pain may vary from neutral (i.e., absence of response) to empathetic, whereas in children's media pain entertains and evokes an amused response and laughter rather than empathy. Portraying pain in the media as fictional and exaggerated facilitated understanding of comedic interactions that were not dangerous and could, therefore, be laughed at. Contrasted with the real-life pain that signals danger and demands attention, pain that was portrayed in the media functioned as light-hearted momentary entertainment requiring no reactions other than laughter.

“*With the younger kids, [pain is used] mostly for humor, right? It's a lot of laughing, like sort of a slapstick approach. Where the pain is funny…Even if the character is being [hurt] in a way that would kill you in a normal life, [the character] just…springs back up”*. (Alice, mother)

Parents differed in their opinions regarding the impact of using pain as entertainment. For some parents, comedic pain was beneficial as it functioned to normalize ubiquitous pain experiences and prevent children from catastrophizing about pain (e.g., “*most things are recoverable from, nothing is ever catastrophic”*, Nina, mother). In contrast, other parents noted that comedic portrayals of pain may be problematic as it may desensitize children to pain in real life. Further, pain as comedy and entertainment failed to draw upon the critically important associations between pain and danger or threat, and pain and concern for oneself or others. Comedic pain violated the social norms regarding responding to another's pain, such that it taught children to disregard and laugh at people experiencing pain, which, in turn, might contribute to the lack of empathy for others' experiencing pain.

“*If somebody falls down and it's played for laughs, it teaches [children] that's funny, and you don't necessarily have to help the person… The right reaction is to laugh at them, not to help”*. (Jillian, mother).

For some parents, there was a fundamental disconnect between media-portrayed- and real-life- pain. For these parents, comedic/entertaining pain in children's media did not and could not impact children's pain experiences or contain any lesson because pain portrayed in media and pain experienced in everyday life belonged to two distinct entities. According to some parents, everyday pain was a highly subjective experience and did not equate to pain portrayed in the media that was shown as an object of entertainment and laughter. Factors that mattered for everyday, real pain (e.g., children's temperament and parents' reactions to children's pain experiences) did not play a role in pain that was portrayed in media.

“*I wouldn't necessarily say that [pain influences children's pain experiences]. In my experience with my children, their pain is always very subjective to them and [embedded] in the moment of what's going on with them. I've never heard them reference a TV show or a movie. It's almost [like] they have two separate worlds.”* (Katie, mother)

Further, for some parents, integrating pain experienced in real life with pain displayed in the media (e.g., portraying pain in shows in a more realistic manner) meant moving away from the core function of children's media—entertainment. Represented as exaggerated and clearly not real, pain in the media gave children permission to laugh at it and be entertained by it. Any realistic features of pain (i.e., depicting any typical physiological [e.g., blood, scratch, bruise] or psychological [e.g., suffering, fear] consequences of pain) would revoke this permission, remind children about the unpleasant and often scary daily experience of pain, and be developmentally inappropriate.

“*When you start portraying the injuries realistically,…you really start moving out of the entertainment. Watching somebody else's pain, not their own pain, it was something you could laugh at cause you knew that it wasn't real”*. (Jack, father)

Taken together, this theme captures parents' perception of pain portrayed as entertaining. In children's media, pain could be entertaining because it was portrayed as unrealistic, fleeting, leading to no lasting consequences, devoid of emotions, and being easily fixed. Another reason for pain being entertaining was its comedic portrayal that, according to parents, normalized pain or, in contrast, desensitized children to pain. Comedic and entertaining, pain in the media was perceived by some parents as entirely different and separate from pain experienced in real life. Any shifts toward a more realistic portrayal of pain in media would remove the entertainment function of children's media and be developmentally inappropriate.

### Theme Two: Valuable Lessons

The second theme captures parents' perspectives about pain portrayals as instilling valuable lessons. As opposed to a plot mechanism that lacks significance in its own right, pain as a lesson was a core, inherently valuable part of the story. It was perceived as eliciting appropriate emotional responses from the characters experiencing pain and empathic reactions from the observing characters. Further, it gave children a point of reference and language for their own painful experiences.

Some parents stated that, in contrast with the entertaining portrayals of pain, realistic portrayals of pain are rare. Some parents highlighted that only certain types of pain (i.e., needle-related, procedural, and illness-related pain) were portrayed accurately, compassionately, and contained helpful lessons for children. The accurate and helpful portrayal of pain included distressing pain-related emotions, as well as coping strategies to help alleviate pain and emotional distress in characters experiencing pain. As some parents observed, the affective aspect of pain was prominently present in realistic pain depictions and contrasted with the emotion-free, entertaining/comedic pain depictions. Thus, as opposed to being a comedic plot mechanism, pain was perceived as a key and complex story component that included, required, and elicited a variety of appropriate emotional reactions (e.g., being scared) and learning opportunities (e.g., learning about causes of pain and ways to deal with pain-related emotions).

“*[The show] tries to be…as real as possible with lessons about how to deal with the emotions pre, during, and post [needle]. I think it gets portrayed in a very accurate way…they talk about some tools and strategies to deal with the emotions that [children are] anticipating.”* (Carl, father)

Another perceived contrast between entertaining pain and pain as valuable lessons unfolds in the social context of pain. Comedic pain elicited laughter and provided entertainment. Parents reflected that when portrayed as a learning experience, pain elicited concern and empathy/compassion for one's own and another person's pain and modeled socially acceptable responses (e.g., seeking or offering help, promoting collaboration and empathy in response to pain). Beyond teaching empathy for pain, media portrayals of pain and children's favorite characters provided children with a frame of reference and appropriate language to express and appropriately respond to their own or another person's pain.

“*It gives them…some language and some experiences that they can then build off of. So, it could be play, it could be having a conversation, sometimes I'll hear what's coming out of the shows echoed back…I think it's another means of giving those [painful experiences] language, a response pattern that is socially acceptable.”* (Kalinda, mother)

Some parents reflected on another lesson relayed through pain portrayal: differences as a function of characters' genders. Parents noted that male characters experienced numerous and severe painful injuries, whereas female characters were more likely to suffer from minor injuries and stomach aches. Further, parents contrasted boy characters' resilience in the presence of physical pain with emotional pain of girl characters who were portrayed as needing and receiving more help. Some parents thought these differences might reflect real-life gender differences in pain experiences. Other parents were concerned that gendered pain portrayal reinforces gender stereotypes, which, in turn, might be internalized by children, impact their self-efficacy, and model differential, and possibly unhelpful, responses to boys vs. girls in pain.

“*Boy characters are rough and tough and laugh it off immediately…The girl characters get…more sympathy for their injuries. I think that also keeps going with the gender roles and stereotypes that we have…even though we're telling ourselves we're not pushing kids into those unhelpful stereotypes, we're still doing that by [showing them] the cartoons”. (Monica, mother)*

In sum, the second theme represents parents' beliefs that the portrayal of pain in media may contain valuable lessons for children. When portrayed accurately (i.e., laden with typical pain-related emotions and eliciting socially acceptable, and empathic responses), pain could provide children with a helpful benchmark and appropriate language for their own painful experiences, as well as model empathy for pain. Yet, the ways in which pain is currently portrayed in children's media might be relaying (unhelpful) gender stereotypes and prompt children to internalize them.

## Discussion

Pain is ubiquitous in both everyday life (16) and in children's popular media (12). Media plays a powerful role in shaping children's development (7). Given the critical role of parents in making decisions about viewing and explaining media content to children, as well as a notable influence that parents have within the context of pediatric pain (23), parental beliefs regarding the portrayal of pain in children's media are important, yet understudied. In this study, we aimed to examine parental beliefs and perceptions of the portrayal of pain in children's popular media. Inductive reflexive thematic analysis revealed two key themes: “entertaining pain” and “valuable lessons”. These themes represent parental views on contrasting portrayals of pain, the function of representing pain in the media, the specific messages that these portrayals comprise, and their relevance and significance (or lack thereof) in children's lives.

The portrayal of pain in children's media was perceived by parents to be entertaining due to two key reasons. First, pain that was portrayed in media was devoid of typical pain characteristics (e.g., facial reactions to pain, pain-related emotions, appropriate social responses). Second, pain that was portrayed either immediately stopped and/or was easily fixed. The absence of portrayals of the socio-emotional facets of pain and its consequences (e.g., some degree of suffering/unpleasantness, the temporal differentiation between injury and pain relief) allowed pain to become a plot mechanism propelling the story, as opposed to being one of the important storylines.

The second reason for an entertaining portrayal of pain was its comedic depiction. In the social context of pain that was portrayed, normative prosocial and empathic responses to pain were replaced with the dismissal of pain and/or characters' amusement and laughter. Pain as comedy and entertainment has always been present in popular culture (24) due to how easily understandable pain and its effects are (24). “A high level of education is not needed to comprehend what is happening when one performer hits another with a piece of wood” (p. 63, 24). Similarly, higher-level cognitive abilities, which are still developing in young children, are needed to appreciate sarcasm and verbal irony (25), but not to appreciate physical comedy. This innate familiarity and understandability of pain may contribute to how normative it is to depict pain as comedy in children's popular media.

Parental opinions regarding the comedic portrayal of pain and light-hearted reactions varied from positive appraisal (e.g., normalizing pain in everyday life, reducing catastrophizing about pain) to concerns regarding the underlying message (e.g., desensitizing children to pain, encouraging unhelpful responses to pain). Importantly, for some parents, pain portrayed in media and pain experienced in real life did not share any common features. Unrealistic, comedic, and, thus, entertaining, pain that is portrayed in children's media did not and should not represent everyday pain, a subjective, unpleasant, and distressing experience. For parents sharing this view, the clear differentiation between real-life pain and pain portrayed in the media reinforced the sole function of pain in media—entertainment.

A contrasting parental perspective on the portrayal of pain focused on pain as instilling valuable lessons for children. Indeed, this is supported by existing research. Children learn about pain by frequently experiencing it themselves (16), observing others in pain (26), and, likely, consuming media (i.e., books, television shows) (8). Exposure to media has been argued to result in short-term (e.g., priming, or partially activating, existing behavioral scripts and imitation of observed behaviors) and long-term effects (e.g., observational learning of societal norms, activation or desensitization of emotional processes, didactic learning) (7, 27). Thus, the portrayal of pain in the media may indeed present a learning opportunity about pain *and* its socio-emotional components.

When depicted accurately and eliciting appropriate emotions and compassionate social reactions from others (e.g., empathy, helping behaviors), pain portrayals in the media were perceived by parents as offering valuable lessons. Empathy and/or compassion toward pain is of particular importance. The developmental trajectory of empathy for pain differs from empathy for other distressing emotions (e.g., sadness). Children aged 18 months (Group 1) and 3 years (Group 2) were similarly distressed by observing another person being sad (14). Yet, when witnessing another person in pain, 18-month-old children expressed *less* concern than children aged 3 years (14). Additional scaffolding and modeling of empathic responses toward another person's/character's pain could support the development of empathy in children.

Parents also observed and reflected on gender differences in how pain was portrayed in popular media. Specifically, parents noticed that boy characters experience more pain associated with physical injuries and exhibit resilience. In contrast, girl characters experience more pain associated with emotional events, are less resilient, and yet receive more help. These observations mirror those of a recent study in which girl characters in children's media, when in pain, were more likely to ask for and receive help, than boy characters who experienced more severe injuries. These differences may be contributing to differential socialization of pain between boys and girls, which, in turn, may contribute to gender differences in pediatric pain (e.g., girls remembering past pain as more intense than boys remember it to be (28), chronic pain being more prevalent in girls than in boys (29)). However, future research is needed to support this.

It is important to consider these findings in a developmental context. Young children's cognitive and socioemotional skills undergo rapid, critical changes during the period between 4 and 6 years of age. According to Piaget's theory of cognitive development, this age period comprises the preoperational stage (30). During the preoperational stage, children form internal representations of the world through their evolving language and mental imagery (30). Children also learn to think symbolically, in other words, they learn to recognize that one thing (i.e., a symbol) represents another thing in addition to itself (30). Given that media is a form of symbolic communication, children start learning that a screen is a symbolic representation of the world around them. Thus, how pain is portrayed in children's popular media may become one of symbolic representations of pain in real life. This conclusion is in direct contradiction with the parental view of pain portrayed in media and pain in real life existing in two distinct and independent worlds. Further, some parents resisted the idea of a more realistic portrayal of pain as it would eliminate pain's entertaining function. This presents an interesting learning opportunity to be employed by parents when discussing television shows and movies with children. When watching a character that is getting repeatedly hurt in a series of comedically portrayed accidents, parents may choose to highlight how entertaining it is on screen, and next discuss the functions and value of pain in real life (e.g., What would it feel like to experience these injuries in real life?; How can the character just get up and immediately recover from their injury?; How come didn't anyone help the character?; How do you think that would make the character feel?; What should others do to help him/her/them feel and get better?). A review of children's learning from fictional sources (e.g., books, video games, movies) highlighted the active role that parents and teacher may need to take in processing media messages (6).

The portrayal of pain as entertainment may be possible as children's media delineates injury (e.g., falls, accidental hits) from pain. Injuries, but not pain, are often depicted in children's shows; typical individual (e.g., nociception, facial expressions of pain, pain-related fear, suffering, interruption of previous activity) and interpersonal (e.g., expressions of compassion, helping behaviors) consequences of injuries are largely absent from the children's media. Facial expression following an injury may be a key factor in subsequent reactions. In a study investigating electrophysiological correlates of adult humor processing, a laughing reaction was triggered if a participant saw a funny, amused, or bewildered facial expression in the victim (31). By contrast, a facial expression of fear or anger elicited empathetic response (31). Therefore, since pain instances portrayed in children's media often lack these emotional responses (12), they may indeed fail to evoke empathy, and rather evoke laughter and amusement, in the viewers (i.e., children).

This study should be viewed in light of limitations that underscore important avenues for future research. First, the study sample was homogenous, with the majority of parents identifying as white, college-educated, and belonging to middle/upper-middle class. Given cultural differences in entertainment and comedy preferences (32), parental beliefs about pain that is portrayed may differ as a function of parents' culture, ethnicity, or education levels. Future research should be conducted with more diverse samples. Second, participating parents were aware that the present research study centered around pain and was conducted by a pain research laboratory within a university. Additionally, parents were primed to think about pain having watched and discussed video clips depicting pain prior to the interview. Parents also discussed the viewed clips with their children prior to the parent interview; children's opinions may have altered parental opinions about pain that is portrayed. Further, parents were not asked whether they or their children had a persistent pain problem; people living with chronic pain may exhibit cognitive biases (e.g., selective attending to pain) (33). Combined, these factors might have resulted in self-edited and/or pain-focused responses. In future studies, blinding parents to the study aims may produce responses that are less focused on pain, which would allow for examination of how frequently parents think about the portrayals of pain, without any external prompts. Additionally, in the future studies, it would be beneficial to collect additional information about parents (e.g., family pain history, parent levels of anxiety and pain catastrophizing, parental general attitudes toward media and children's media use) as these variables may influences parental perceptions and beliefs about pain that is portrayed. Third, the present study focused solely on the portrayal of pain. Given the key differences in how parents and children talk about past pain vs. sadness or fear (34, 35) and how children react to pain and sadness in other people (14), it will be important to examine how parents perceive the portrayal of pain when contrasted to the portrayal of negative emotions (e.g., sadness, fear, anger). Finally, the study examined solely on parental perspectives regarding the portrayal of pain in children's popular media. To our knowledge, this is the first study that recruited an approximately equal number of mothers and fathers to allow for a balanced representation of parental beliefs. However, future research should include children's opinions and understanding of pain that is portrayed and examine these perceptions across developmental trajectories.

Pain is as common in everyday life as it is on the screens. In watching popular media, children witness multiple instances of injuries with and without pain, and an array of pain behaviors (12). Given the key role of parents in both pediatric pain and children's media consumption, parental beliefs regarding how pain is portrayed in children's popular shows and movies are important to consider. The present study identified two contrasting themes in parental beliefs and attitudes about pain that is portrayed: “entertaining pain” and “valuable lessons”. These findings contribute to our understanding of how children's media messages concerning pain are understood and interpreted by parents. The findings also highlight a possible learning opportunity for children to be used by parents and/or educators. Watching media that portray pain as entertaining may be followed by parent/educator-child discussions regarding the differences between pain in real life and pain portrayed in media, as well as functions and value of pain in real life.

## Data Availability Statement

The raw data supporting the conclusions of this article will be made available by the authors, without undue reservation.

## Ethics Statement

The studies involving human participants were reviewed and approved by Conjoint Faculties Research Ethics Board (CFREB) of the University of Calgary. Written informed consent to participate in this study was provided by the participants' legal guardian/next of kin.

## Author Contributions

All authors listed have made a substantial, direct, and intellectual contribution to the work and approved it for publication.

## Funding

This work was supported by the Alberta Children's Hospital Foundation. MN holds the Killam Memorial Emerging Leader Chair and is funded by the Killam Trusts, Canadian Institutes of Health Research, Social Sciences and Humanities Research Council, Alberta Children's Hospital Foundation, Chronic Pain Network, and the Chronic Pain Center of Excellence for Canadian Veterans. MP was supported by the Alberta Strategy for Patient-Oriented Research Graduate Studentship, the University of Calgary Eyes High Doctoral Scholarship, Alberta Innovates Graduate Studentship in Health Innovation, Frederick Banting and Charles Best Canada Graduate Scholarships, and Izaak Walton Killam Doctoral Scholarship. GM and SW are supported by a Leadership Investigator Grant to GM from the National Health & Medical Research Council of Australia (ID 1178444).

## Conflict of Interest

GM has received support for pain-related work from: Reality Health, Connect Health UK, Kaiser Permanente, AIA Australia, Workers' Compensation Boards and professional sporting organisations in Australia, Europe, South and North America. Professional and scientific bodies have reimbursed him for travel costs related to presentation of research on pain at scientific conferences/symposia. He has received speaker fees for lectures on pain and rehabilitation. He receives royalties for books on pain and pain education. None of these bodies had any role in the current work. The remaining authors declare that the research was conducted in the absence of any commercial or financial relationships that could be construed as a potential conflict of interest.

## Publisher's Note

All claims expressed in this article are solely those of the authors and do not necessarily represent those of their affiliated organizations, or those of the publisher, the editors and the reviewers. Any product that may be evaluated in this article, or claim that may be made by its manufacturer, is not guaranteed or endorsed by the publisher.
